# microRNA-181a-5p impedes the proliferation, migration, and invasion of retinoblastoma cells by targeting the NRAS proto-oncogene

**DOI:** 10.1016/j.clinsp.2022.100026

**Published:** 2022-03-24

**Authors:** Ming Ouyang, Guiqin Liu, Cheng Xiong, Jing Rao

**Affiliations:** a Shenzhen Eye Hospital, Jinan University, China; b Shenzhen Eye Hospital, Shenzhen Eye Institute, Jinan University, School of Optometry, Shenzhen University, China

**Keywords:** miR-181a-5p, NRAS, Retinoblastoma

## Abstract

**Objectives:**

Accumulating research have reported that microRNAs (miRNAs) play important roles in Retinoblastoma (RB). Nonetheless, the function and underlying mechanism of miR-181a-5p in RB remain ambiguous.

**Methods:**

The relative expression levels of miR-181a-5p and NRAS mRNA were detected by quantitative Reverse Transcription-Polymerase Chain Reaction (qRT-PCR). RB cell proliferation was measured using the Cell Counting Kit-8 (CCK-8) and 5′-Bromo-2′-deoxyuridine (BrdU) assays. Transwell assays and flow cytometry were performed to detect the migration, invasion, and apoptosis of RB cells. The interaction between miR-181a-5p and NRAS was explored using luciferase experiments, western blotting, and qRT-PCR.

**Results:**

miR-181a-5p expression was found to be decreased in RB tissues and cell lines, and its expression was correlated with unfavorable pathological features of the patients. *In vitro* experiments revealed that miR-181a-5p reduced RB cell proliferation, migration, and invasion while enhancing apoptosis. Further research confirmed that NRAS is a direct target of miR-181a-5p. miR-181a-5p inhibited NRAS expression at both the mRNA and protein levels. Co-transfection of pcDNA-NRAS or NRAS small interfering RNA (siRNA) reversed the effects of miR-181a-5p mimics or miR-181a-5p inhibitors on RB cells.

**Conclusion:**

miR-181a-5p was significantly downregulated during the development of RB, and it suppressed the malignant behaviors of RB cells by targeting NRAS.

## Highlights

• miR-181a-5p expression was found to be decreased in retinoblastoma tissues and cell lines.
• miR-181a-5p reduced retinoblastoma cell proliferation, migration, and invasion while enhancing apoptosis.
• NRAS is a direct target of miR-181a-5p.

## Introduction

Retinoblastoma (RB) is the most common intraocular malignancy in children, with worldwide morbidity of approximately 1/15,000 to 1/20,000.^1^ The treatment of RB has greatly improved in the past few decades, and early diagnosis can also effectively improve the prognosis of RB.^1^^,^^2^ However, in less developed areas, the treatment effect of RB is still unsatisfactory due to the delay in diagnosis.^2^ Clarifying the underlying mechanism of RB progression is of great significance to further improve the diagnosis and treatment of RB.

The abnormal expression of microRNAs (miRNAs) in tumors and their effects on the biological behavior of tumor cells have attracted a lot of attention. Targeted therapy based on miRNA is expected to become a novel treatment strategy for tumors, including RB.^3^ For instance, miR-124 was found to be significantly downregulated in RB tissues and cell lines, and *in vitro* assays revealed that miR-124 suppresses the proliferation, migration, and invasion of RB cells and induces apoptosis by targeting Signal Transducer and Activator of Transcription (STAT)-3;^4^ miR-25-3p is found to be significantly increased in RB tissues and cell lines. It not only promotes the growth, migration, and invasion of the RB cell line WERI-Rb-1, but also facilitates the Epithelial-Mesenchymal Transition (EMT) by regulating the Phosphatase and Tensin homolog (PTEN)/serine-threonine kinase (Akt) signaling pathway.^5^ miR-181a-5p has also been reported to affect the progression of diverse tumors. For instance, compared with Gastric Cancer (GC) tissues without distant metastasis, miR-181a-5p expression is remarkably increased in GC tissues with distant metastasis, and miR-181a-5p overexpression remarkably enhances the proliferation, migration, and invasion of GC cells via the targeted downregulation of the Ras Association domain Family member-6 (RASSF6).^6^ However, in Non-Small Cell Lung Cancer (NSCLC) tissues and cell lines, miR-181a-5p expression is remarkably reduced, and after transfection of miR-181a-5p mimics into the NSCLC cell line, A549, both proliferation and migration are markedly reduced.^7^ Compared with the normal retinal tissues, miR-181a expression is notably reduced in RB tissues.^8^ Nonetheless, the specific function of miR-181a-5p in RB remains largely unknown.

The RAS gene family members, including NRAS proto-oncogene and GTPase (NRAS), are among the most frequently activated oncogenes in cancers. NRAS activates various signaling pathways, such as the Phosphoinositide 3-Kinase (PI3K)/AKT and Nuclear Factor-kappa-B (NF-κB) pathways, to enhance the proliferation, migration, and invasion of tumor cells.^9^^,^^10^ For example, in melanoma, NRAS expression is significantly increased, and miR-145-5p inhibits the activation of Mitogen-Activated Protein Kinase (MAPK) and PI3K/AKT signaling pathways by repressing NRAS, thereby inhibiting melanoma cell proliferation, migration, and invasion.^10^ In colorectal cancer, NRAS is decreased significantly in cancerous tissues, and miR-144 can inhibit NRAS expression as well as the growth and migration of SW480 cells.^11^ NRAS expression is remarkably increased in RB tissues and cell lines, and silencing NRAS with a small interfering RNA (siRNA) markedly suppressed the proliferation, migration, and invasion of RB cells *in vitro* and repressed the tumor growth *in vivo*.^12^ Nevertheless, the upstream regulatory mechanisms of NRAS have not yet been fully elucidated.

In the present study, the authors found that miR-181a-5p expression was markedly downregulated in RB tissues and cell lines, and there was a potential binding site between miR-181a-5p and the 3′-Untranslated Region (UTR) of NRAS. Therefore, the authors hypothesized that the miR-181a-5p/NRAS axis could exert certain effects on the development of RB.

## Materials and methods

### Ethics statement

This study was approved by the Ethics Committee of the Shenzhen Eye Hospital (Futian District, Shenzhen, Guangdong Province, China) (201401013). Written informed consent was obtained from each participant. All protocols were conducted in accordance with the principles of the Declaration of Helsinki.

### Clinical samples

The authors obtained 33 RB tissue samples and 12 normal tissue samples from Shenzhen Eye Hospital from February 2014 to November 2018. Tissues were collected from patients with RB who did not receive any anti-cancer therapy before surgical resection. All 33 patients with RB were histopathologically identified and staged according to the American Joint Committee on Cancer (AJCC) staging system. Normal retinas were obtained from 12 children who died of non-ophthalmic diseases. All guardians of the patients provided informed consent. This study was approved by the Ethics Committee of the Shenzhen Eye Hospital.

### Cell culture and transfection

Human normal retinal pigment epithelial cells (ARPE-19) and RB cell lines (HXO-RB44, SO-Rb50, Y79, and WERI-RB-1) were procured from the American Type Culture Collection (ATCC, Rockville, MD, USA) or China Center for Type Culture Collection (CCTCC, Wuhan, China). Cells were cultured in the Dulbecco's Modified Eagle's Medium (DMEM) (Gibco, Grand Island, NY, USA) supplemented with 10% Fetal Bovine Serum (FBS) (Gibco, Grand Island, NY, USA), 100 U/mL penicillin, and 100 μg/mL streptomycin (Gibco, Grand Island, NY, USA). The cells were maintained in a humidified incubator at 5% Carbon Dioxide (CO_2_) and 37°C. Cells in the logarithmic growth phase were harvested for subsequent experiments.

NRAS overexpression plasmid (pcDNA3.1-NRAS), NRAS siRNA, miR-181a-5p mimics, miR-181a-5p inhibitor, and their negative controls were purchased from GenePharma (Shanghai, China). Following the instructions, Lipofectamine® 3000 (Invitrogen, Carlsbad, CA, USA) was used to transfect the NRAS-overexpressing plasmid and miR-181a-5p inhibitors into the SO-Rb50 cell line; NRAS siRNA and miR-181a-5p mimics were transfected into the Y79 cell line.

### Quantitative reverse transcription-polymerase chain reaction (qRT-PCR)

According to the instructions, TRIzol reagent (Invitrogen, Carlsbad, CA, USA) was used to extract the total RNA of tissues and cells. The concentration and purity of the extracted RNA were measured using NanoDrop 2000 (Thermo Fisher, Asheville, NC, USA), and the total RNA was reverse transcribed into cDNA using a Transcriptor First Strand cDNA Synthesis Kit (Roche, Mannheim, Germany). SYBR Premix Ex Taq™ (TaKaRa, Otsu, Shiga, Japan) was used, with cDNA as a template for PCR amplification. U6 and glyceraldehyde 3-phosphate dehydrogenase (GAPDH) were used as internal references, and their relative expression was calculated using the 2^−ΔΔCT^ method. The sequences of each primer are shown in Table 1.

**Table 1:** The Polymerase Chain Reaction (PCR) primers used in this research study.

Name	Primer sequences
miR-181a-5p	Forward: 5′-GCCGAACATTCAACGCTGTCG-3′
	Reverse: 5′-GTGCAGGGTCCGAGGT-3′
NRAS	Forward: 5′-ATGAGGACAGGCGAAGGC-3′
	Reverse: 5′-TGAGTCCCATCATCACTGCTG-3′
U6	Forward: 5′-CTCGCTTCGGCAGCACA-3′
	Reverse: 5′-AACGCTTCACGAATTTGCGT-3′
GAPDH	Forward: 5′-ATGGAAATCCCATCACCATCTT-3′
	Reverse: 5′-CGCCCCACTTGATTTTGG-3′

### Cell counting kit-8 (CCK-8) assay

RB cells in the logarithmic growth phase were trypsinized and seeded in a 96-well plate with 100 μL of cell suspension per well (2 × 10^3^cells per well). The 96-well plates were then placed in an incubator to continue the culture. After 24h, 10 μL of CCK-8 solution (Beyotime, Shanghai, China) was added to each well, and the cells were incubated for 1h. After the culture was terminated, the 96-well plate was placed in a microplate reader, and the absorbance (optical density, OD value) of each well was measured at a wavelength of 450 nm. Thereafter, the OD values of the cells were measured at 48h, 72h, and 96h.

### 5′-Bromo-2′-deoxyuridine (BrdU) assay

A BrdU cell proliferation detection kit (Abcam, Shanghai, China) was used to detect the RB cell proliferation. RB cells in the logarithmic growth phase were prepared into a single-cell suspension and seeded in 24-well plates (1 × 10^5^ cells/well). The BrdU labeling reagent was added according to the manufacturer's instructions, and the cells were incubated for 24h. After that, the cells were fixed and incubated with an anti-BrdU antibody (Abcam, Shanghai, China). Next, the cells were stained with 4′, 6-Diamidino-2-Phenylindole (DAPI) staining solution (Abcam, Shanghai, China). The number of BrdU-positive cells and the total number of DAPI-positive cells in three fields were randomly selected and counted under a fluorescence microscope (Olympus, Tokyo, Japan). Cell proliferation rate = number of BrdU-positive cells/DAPI-positive cells

### Transwell assay

A Transwell assay was conducted to detect the RB cell migration and invasion. The cells of each group were prepared into cell suspensions (1 × 10^5^cells/mL) in serum-free DMEM. Subsequently, 200 μL of cell suspension was added to the upper chamber of a two-chamber Transwell system (Corning, Shanghai, China), and 500 μL of medium containing 20% FBS was added to the lower chamber. After culturing at 37°C for 24h, the cells remaining on the upper surface of the membrane were removed, and the cells on the lower surface of the membrane were fixed with formaldehyde, washed with Phosphate-Buffered Saline (PBS), stained with the crystal violet solution (Beyotime, Shangai, China), washed with tap water after staining, and finally counted under a microscope (Nikon, Tokyo, China). Matrigel (Millipore, Billerica, MA, USA) was used to cover the membrane before the cells were inoculated in the invasion assay, while in the migration assay, Matrigel was not used.

### Flow cytometry

The cells were digested with trypsin and collected by centrifugation (20 × g). The apoptotic rate of the harvested cells was detected using an Annexin V-FITC-Propidium Iodide (PI) Apoptosis Detection Kit (Biosharp, Hefei, China). In brief, in each group, approximately 1 × 10^5^ cells were harvested and washed twice with PBS. Pre-cooled PBS (400 μL) was added, and then 10 μL of Annexin V-FITC staining solution and 5 μL of PI staining solution were added. After incubation in the dark at 4°C for 1h, apoptosis was immediately measured using a flow cytometer (BD Bioscience, San Jose, CA, USA).

### Dual-luciferase experiment

The NRAS sequence was amplified and inserted into the plucGLO plasmid (Promega, Fitchburg, WI, USA) downstream of the luciferase gene to obtain the Wild-Type (WT) reporter pmir-NRAS-WT. To obtain the Mutant (MUT) luciferase reporter pmir-NRAS-MUT, the putative binding site for miR-181a-5p in the NRAS sequence was mutated using the GeneArt™ Site-Directed Mutagenesis PLUS System (cat. no. A14604; Thermo Fisher Scientific, Inc., Waltham, MA, USA). pmir-NRAS-WT, pmir-NRAS-MUT, and miR-181a-5p mimics and mimic-NC were co-transfected into SO-Rb50 or Y79 cells using Lipofectamine® 3000 (Invitrogen, Carlsbad, CA, USA). Then, 48h after transfection, the luciferase activity of the cells in each group was analyzed using the Dual-Luciferase Reporter Assay System (GenePharma, Shanghai, China). The firefly luciferase activity in each group was detected, and Renilla luciferase activity was used as a control.

### Western blotting

RB cells were lysed with the Radioimmunoprecipitation Assay (RIPA) buffer (Beyotime, Shanghai, China), and protein samples were prepared. Protein concentration was measured using a bicinchoninic acid kit (Beyotime, Shanghai, China). Fifteen microliters of protein samples from each group were separated using sodium dodecyl sulfate-polyacrylamide gel electrophoresis and then transferred to Polyvinylidene Fluoride (PVDF) membranes (Millipore, Bedford, MA, USA). After blocking with 5% skimmed milk, the membrane was incubated with a primary antibody (anti-NRAS antibody, rabbit anti-human polyclonal antibody, 1:1000, ab154291; Abcam, Shanghai, China) at 4°C overnight and washed with Tris-buffered saline with Tween 20 (TBST, Beyond, Shangai, China). After that, the membrane was incubated with the secondary antibody (goat anti-rabbit IgG, 1:2000, ab205718; Abcam, Shanghai, China) at 25°C for 2h. Protein bands were developed using an ECL kit (Amersham Pharmacia Biotech, Little Chalfont, UK), and the images were captured using an ImageQuant LAS4000 miniature biomolecular imager (GE Healthcare, Chicago, IL, USA). GAPDH was used as an internal reference. ImageJ (v.1.8.0 NIH, Bethesda, MD, USA) was used for the quantitative analysis of the gray values for the protein bands.

### Statistical analysis

SPSS v.23.0 (SPSS Inc., Chicago, IL, USA) was used for the statistical analysis. Measurement data are expressed as the mean ± standard deviation (x±s). The means of two groups of samples were compared using the *t*-test. The difference in composition ratio between groups was tested by the Chi-Square test, and p < 0.05 was used to indicate statistical significance.

## Results

### miR-181a-5p expression is remarkably downregulated in RB and associated with its pathological features

**Fig. 1:**
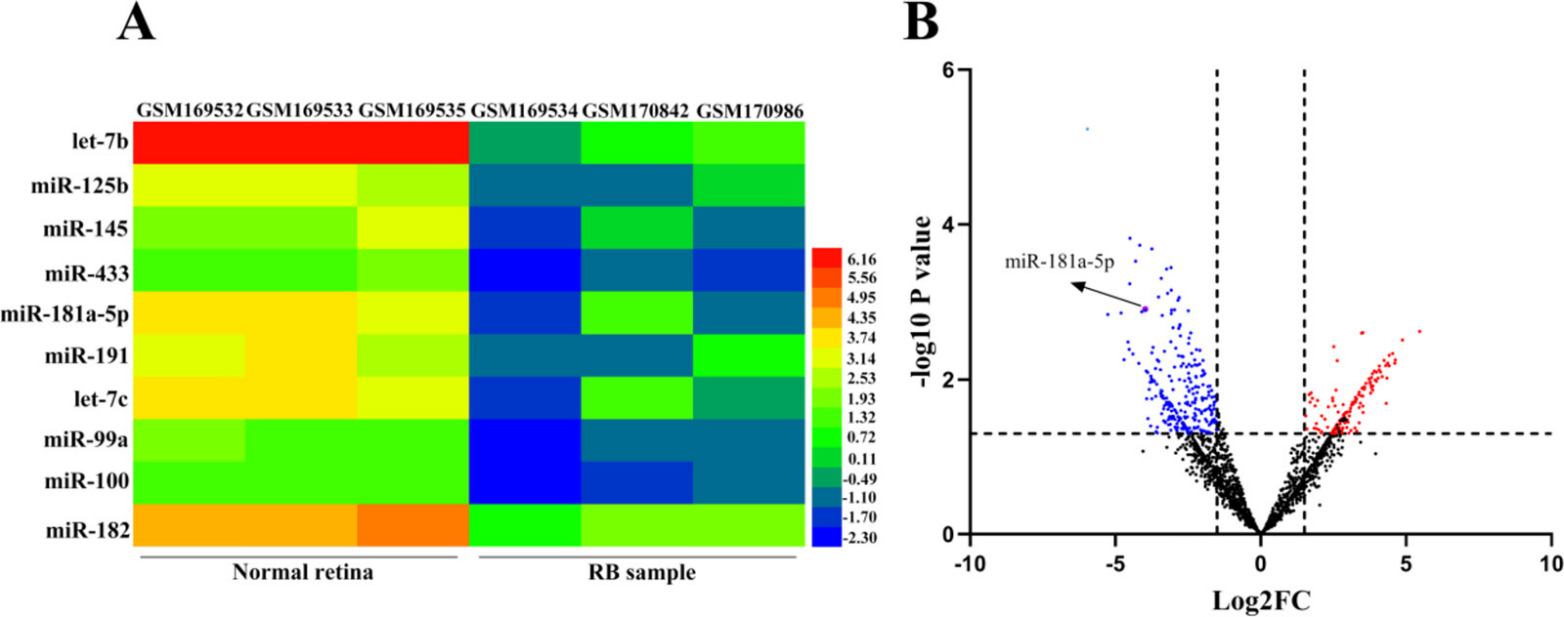
The microarray dataset, GSE7072, is used to screen out the abnormally expressed microRNAs (miRNAs) in Retinoblastoma (RB) tissues and normal tissues. (A) Heat map showing 10 downregulated miRNAs with the most significant statistical differences (log2 fold change < -1 and p < 0.05) between three cases of normal retina samples and three cases of RB samples in GSE7072. Red indicates that the miRNAs are highly expressed in the sample, and blue indicates that the miRNAs are lowly expressed in the sample. (B) Volcano plot showing the differentially expressed miRNAs between three normal retina samples and three RB samples in GSE7072 (log2 fold change > 1 and p < 0.05). Blue represents the downregulated miRNAs, and red represents the upregulated miRNAs. Black represents the miRNAs with no significant differences between RB and normal tissues.

**Fig. 2:**
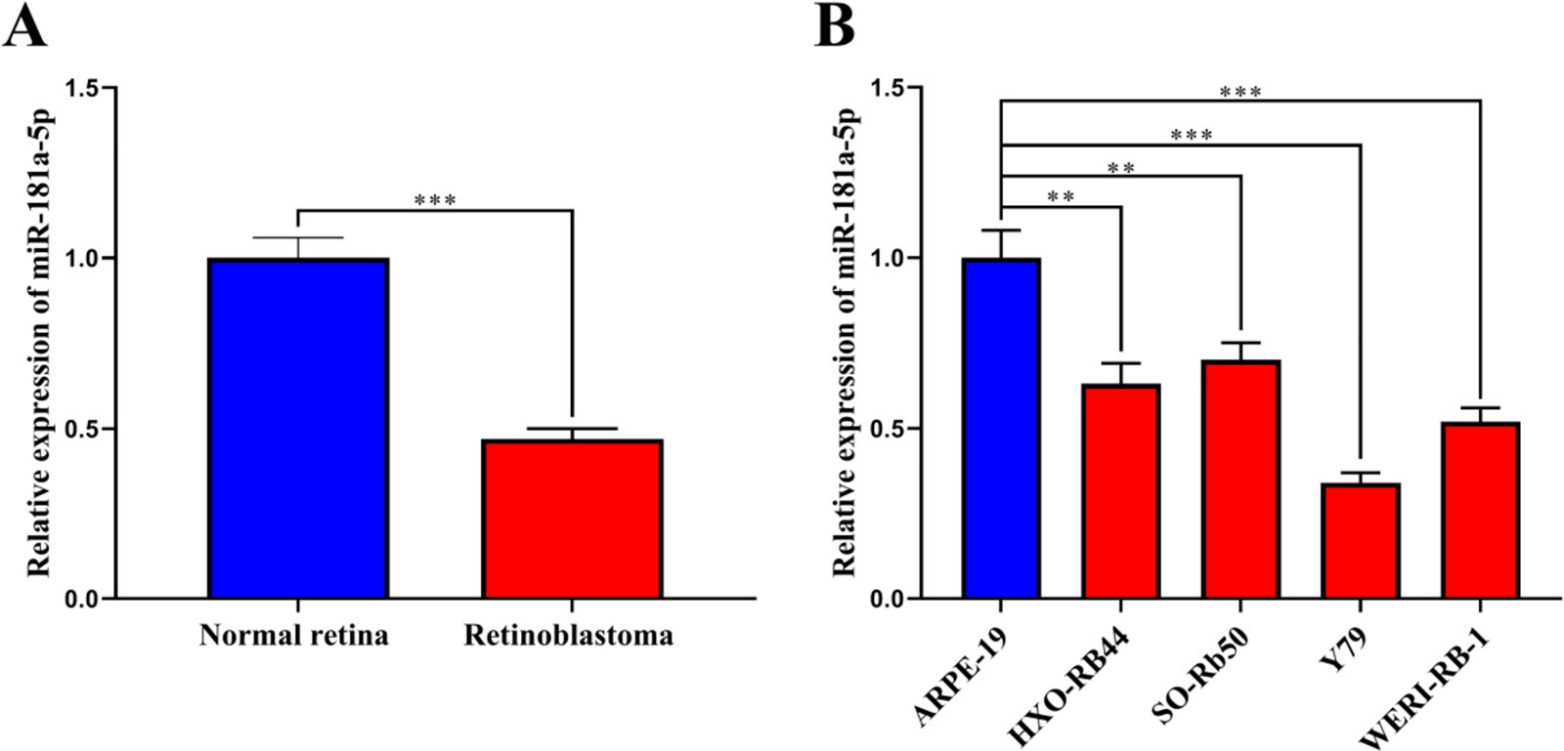
MiR-181a-5p is downregulated in RB tissues and cell lines. (A) Quantitative reverse Transcription-Polymerase Chain Reaction (qRT-PCR) showed that miR-181a-5p was significantly downregulated in RB tissues compared with the normal retinal tissues. (B) qRT-PCR showed that miR-181a-5p was significantly downregulated in RB cell lines compared to the normal retinal pigment epithelial cells. The expression of miR-181a-5p was normalized to that of the U6 small nuclear RNA (snRNA). The data are shown as the mean ± standard deviation from three independent experiments. **p < 0.01 and ***p < 0.001.

First, the authors analyzed the publicly available miRNA expression profile dataset, GSE7072. In this dataset, total RNA samples from three cases of RB samples were profiled for a panel of 160 human miRNAs, and three samples of normal retina tissues were used as controls. It was revealed that miR-181a-5p was remarkably downregulated in RB tissue (in comparison with that in normal retinal tissue) (Fig. 1A-B). The authors hypothesized that miR-181a-5p exerts a tumor-suppressing effect in the development of RB. The authors detected miR-181a-5p expression in RB tissues by qRT-PCR, and the findings showed that miR-181a-5p expression was remarkably downregulated in RB tissues compared to that in normal retinal tissues (Fig. 2A), which was consistent with the data in GSE7072. Furthermore, it was revealed that miR-181a-5p expression in the RB cell lines HXO-RB44, SO-Rb50, Y79, and WERI-RB-1 was notably lower than that in normal retinal pigment epithelial ARPE-19 cells (Fig. 2B). Furthermore, the authors explored the association between miR-181a-5p and the characteristics of patients with RB. The data showed that miR-181a-5p expression was not significantly associated with patient sex and age, but was markedly correlated with tumor aggressiveness, tumor size, and clinical stage (Table 2).

**Table 2:** Correlation between microRNA (miR)-181a-5p expression and the characteristics of RB patients.

Characteristics	All cases	Expression of miR-181a-5p		p-value
		High expression	Low expression	
	33			
Sex				
Male	18	10	8	0.491
Female	15	6	9	
Age(years)				
≥5	13	8	5	0.481
<5	20	9	11	
Invasion				
Non-invasive	22	17	5	0.009
Invasive	11	3	8	
Size (mm)				
≥3	17	6	11	0.037
<3	16	12	4	
IIRC stage				
Early stage (A, B, C)	14	11	3	0.033
Advanced stage (D, E)	19	7	12	

IIRC, Intraocular International Retinoblastoma Classification.

### miR-181a-5p restrains the proliferation, migration, and invasion of RB

**Fig. 3:**
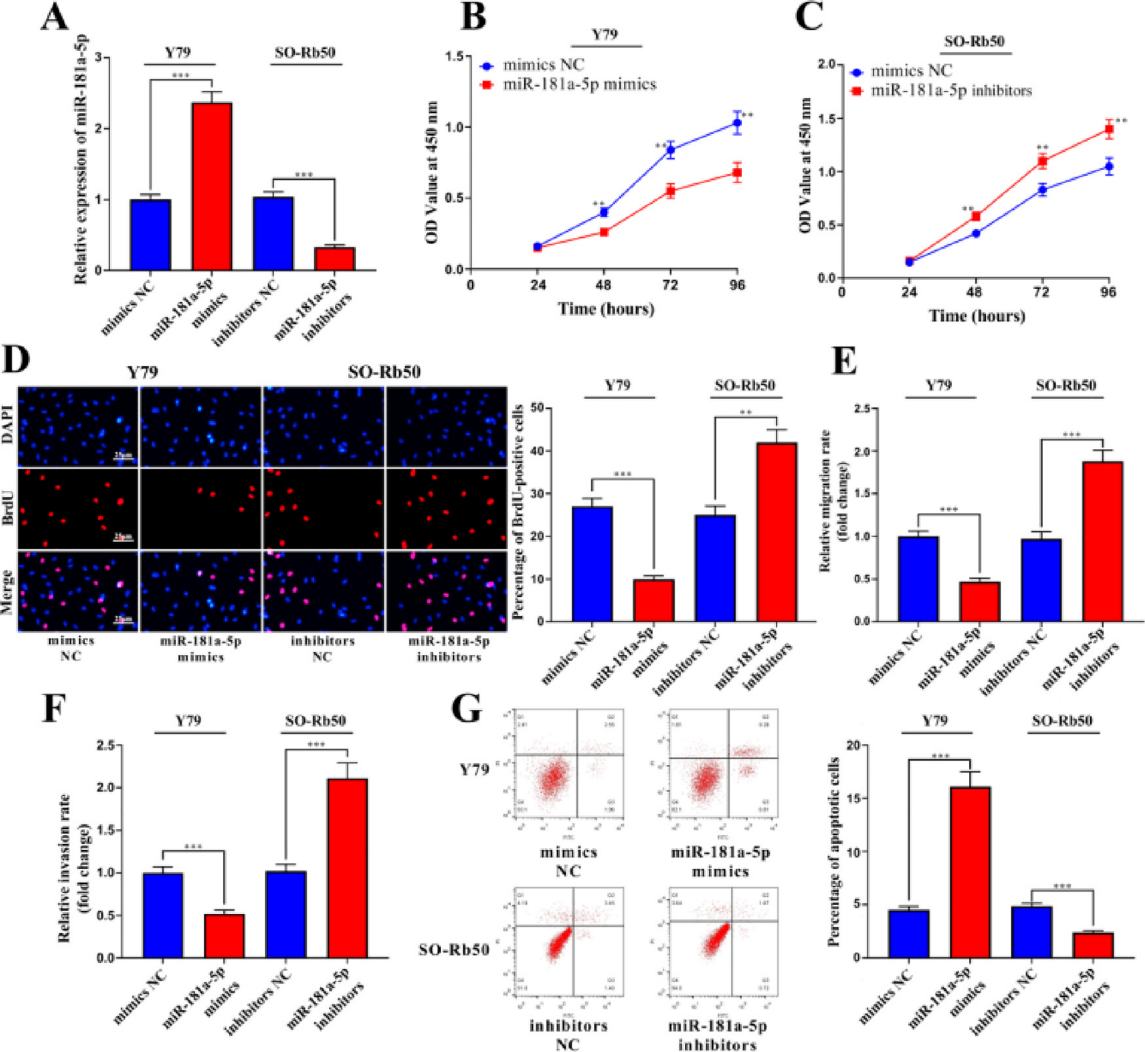
MiR-181a-5p inhibits RB cell proliferation and metastasis and induces cell apoptosis. (A) qRT-PCR showed that miR-181a-5p was upregulated in Y79 cells transfected with miR-181a-5p mimics and downregulated in SO-RB50 cells transfected with miR-181a-5p inhibitors. (B-D) Cell Counting Kit 8 (CCK-8) and 5′-bromo-2′-deoxyuridine (BrdU) assays showed that miR-181a-5p mimics inhibited the cell proliferation, while miR-181a-5p inhibitors promoted the cell proliferation. (E-F) Transwell assay showed that miR-181a-5p mimics inhibited the migration and invasion of Y79 cells, while miR-181a-5p inhibitors promoted the migration and invasion of SO-RB50 cells (magnification:  × 200; bar: 25 μm). (G) Flow cytometry showed that miR-181a-5p mimics promoted apoptosis in Y79 cells, while miR-181a-5p inhibitors inhibited the apoptosis of SO-RB50 cells. Mimics NC indicates the negative control of miR-181a-5p mimics, and inhibitor NC indicates the negative control of miR-181a-5p inhibitors. The data are shown as the mean±standard deviation from three independent experiments. **p < 0.01 and ***p < 0.001.

miR-181a-5p mimics and miR-181a-5p inhibitors were transfected into Y79 cells and SO-Rb50 cells, respectively, to probe the biological functions of miR-181a-5p in RB cells, and transfection efficiency was measured by qRT-PCR (Fig. 3A). CCK-8 indicated that miR-181a-5p markedly attenuated the proliferation of RB cells (Fig. 3B-C), and BrdU experiments further confirmed this conclusion (Fig. 3D). Through Transwell assay, the authors found that miR-181a-5p overexpression remarkably inhibited the migration and invasion of Y79 cells, but the migration and invasion of SO-Rb50 cells were further enhanced after transfection with miR-181a-5p inhibitors (Fig. 3E-F). Flow cytometry suggested that miR-181a-5p overexpression markedly increased the apoptosis of Y79 cells, whereas the opposite phenomenon was observed in the miR-181a-5p inhibitor group (Fig. 3G). These data implied that miR-181a-5p had an inhibitory effect on the malignancy of RB cells.

### NRAS is one of the downstream targets of miR-181a-5p

**Fig. 4:**
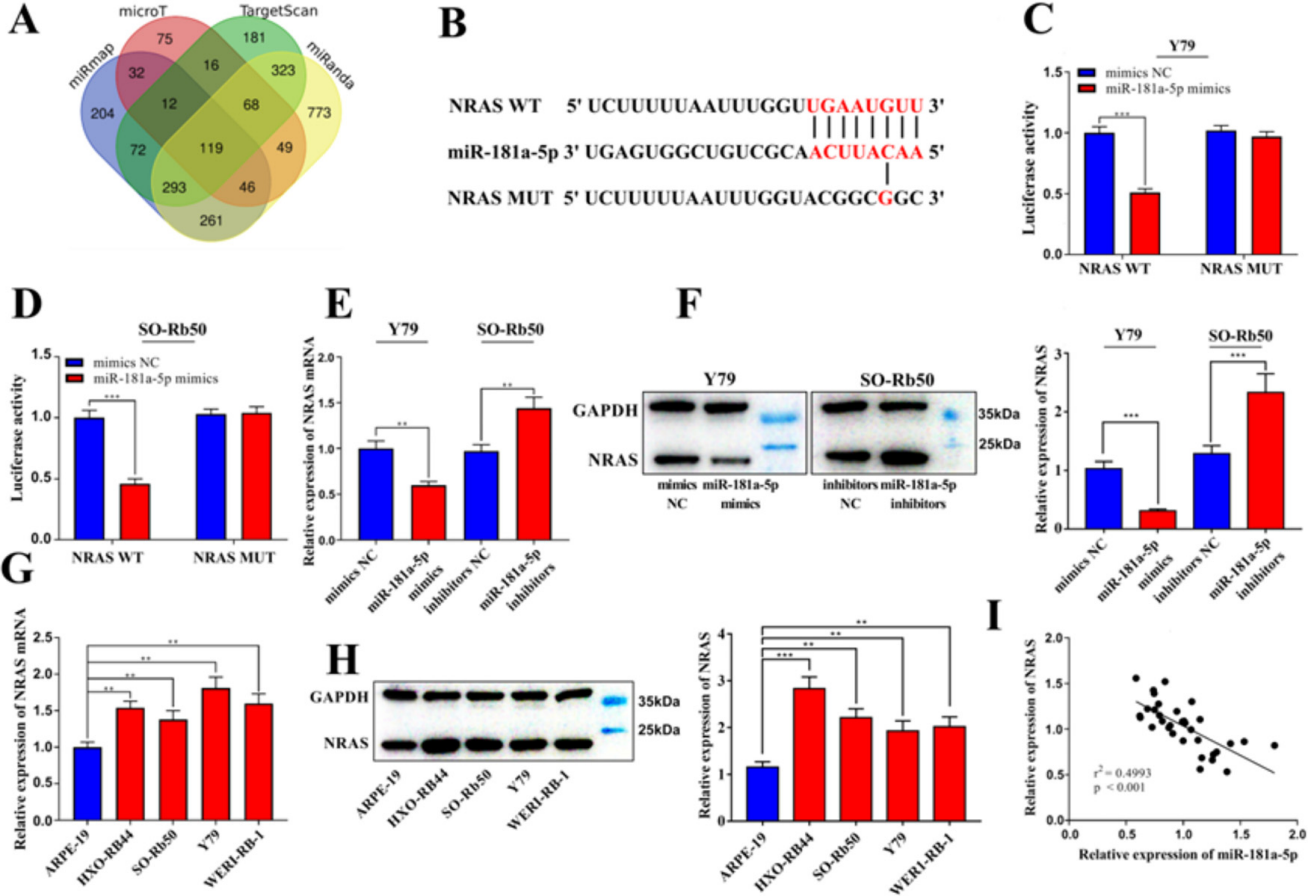
NRAS is a target gene of miR-181a-5p. (A-B) TargetScan, miRanda, miRmap, and microT databases were used to predict the downstream target genes of miR-181a-5p. (C-D) Dual-luciferase reporter gene assay verified that miR-181a-5p could combine with the 3′-Untranslated Region (UTR) of NRAS. (E-F) qRT-PCR and western blotting verified that the expression levels of NRAS mRNA and protein were downregulated in Y79 cells transfected with miR-181a-5p mimics and upregulated in SO-Rb50 cells transfected with miR-181a-5p inhibitors. (G-H) qRT-PCR and western blotting assays showed that the expression levels of NRAS mRNA and protein were significantly upregulated in RB cell lines compared to the ARPE-19 cells. (I) Correlation analysis showed that miR-181a-5p expression was negatively correlated with NRAS expression in RB tissues. Protein bands were quantified by comparison with the endogenous control, Glyceraldehyde 3-Phosphate Dehydrogenase (GAPDH). The data are shown as the mean ± standard deviation from three independent experiments. ** p < 0.01 and *** p < 0.001.

The downstream targets of miR-181a-5p were predicted using four databases, *i.e.*, miRmap, microT, TargetScan, and miRanda (Fig. 4A). It was found that all four databases predicted 119 candidates, and NRAS was one of them (Fig. 4B). Dual-luciferase reporter gene assay revealed that transfection of miR-181a-5p mimics significantly reduced the luciferase activity of the WT NRAS reporter (Fig. 4C-D). qRT-PCR and western blotting indicated that exogenous miR-181a-5p markedly repressed NRAS expression at both the mRNA and protein expression levels while inhibiting miR-181a-5p had the opposite effects (Fig. 4E-F). Additionally, significant increases in NRAS mRNA and protein expression were observed in HXO-RB44, SO-Rb50, Y79, and WERI-RB-1 cell lines compared to those in ARPE-19 cells (Fig. 4G-H). Additionally, in 33 RB samples, a negative correlation was found between NRAS expression and miR-181a-5p expression (Fig. 4I). The above results confirmed that NRAS was a target gene of miR-181a-5p and was suppressed by it.

### NRAS reverses the biological effects of miR-181a-5p on RB cells

**Fig. 5:**
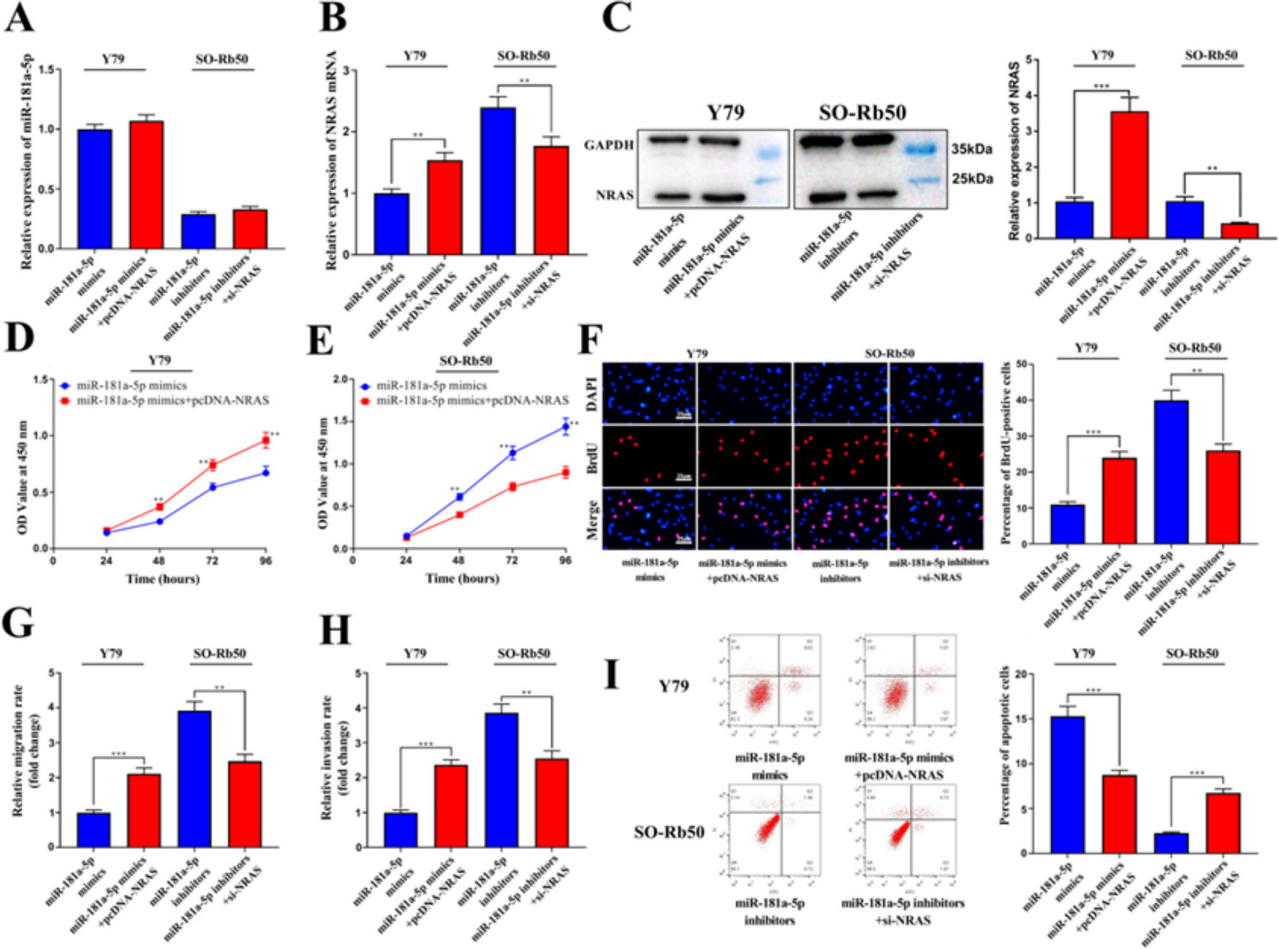
miR-181a-5p exerts an anti-tumor effect in RB by regulating NRAS. (A-C) qRT-PCR and western blotting assays showed that the expression levels of NRAS mRNA and protein were upregulated in the miR-181a-5p mimics+pcDNA-NRAS group compared with the miR-181a-5p mimics group and downregulated in the miR-181a-5p inhibtors+si-NRAS group compared with the miR-181a-5p inhibitor group, while miR-181a-5p expression showed no significant difference. (D-F) CCK-8 and BrdU assays showed that cell proliferation was promoted in the miR-181a-5p mimics+pcDNA-NRAS group compared with the miR-181a-5p mimics group and inhibited in the miR-181a-5p inhibitors+si-NRAS group compared with the miR-181a-5p inhibitor group. (G-H) Transwell assay showed that the migration and invasion of RB cells in the miR-181a-5p mimics+pcDNA-NRAS group was promoted compared with the miR-181a-5p mimics group and inhibited in the miR-181a-5p inhibitors+si-NRAS group compared with the miR-181a-5p inhibitor group (magnification:  × 200; bar: 25 μm). (I) Flow cytometry showed that the apoptosis of RB cells in the miR-181a-5p mimics+pcDNA-NRAS group was inhibited compared with the miR-181a-5p mimics group and promoted in the miR-181a-5p inhibitors+si-NRAS group compared with the miR-181a-5p inhibitor group. Protein bands were quantified by comparison with the endogenous control GAPDH. The data are shown as the mean±standard deviation from three independent experiments. ** p < 0.01 and *** p < 0.001.

To confirm that the function of miR-181-5p is dependent on NRAS, miR-181a-5p mimics and pcDNA-NRAS were co-transfected into Y79 cells, and miR-181a-5p inhibitors and NRAS siRNA were co-transfected into SO-Rb50 cell lines (Fig. 5A-C). Through CCK-8 and BrdU assays, it was revealed that the role of miR-181a-5p mimics in inhibiting the proliferation of Y79 cells was reversed by the restoration of NRAS, and the effects of miR-181a-5p inhibitors on enhancing SO-Rb50 cell proliferation were also abolished by NRAS knockdown (Fig. 5D-F). Transwell assays showed that the effects of miR-181a-5p mimics on regulating the migration and invasion of RB cells were also mediated by its regulatory function on NRAS (Fig. 5G-H). It was also revealed that compared with the miR-181a-5p mimics group, the apoptosis of RB cells was remarkably reduced in the miR-181a-5p mimics+pcDNA-NRAS group, and the apoptosis was remarkably higher in the miR-181a-5p inhibitor+si-NRAS group than that in the miR-181a-5p group (Fig. 5I).

## Discussion

A large number of non-coding RNAs (ncRNAs) are important regulatory factors in various biological processes related to cancer. Clarifying the role of ncRNAs in cancer biology can provide new insights into the diagnosis and treatment of RB.^13^ Through gain-of-function and loss-of-function experiments, the authors found that miR-181a-5p was a regulator of RB progression by suppressing NRAS expression.

The present work revealed that miR-181a-5p expression was markedly downregulated in RB tissues and multiple RB cell lines, which is consistent with the results obtained in the dataset GSE7072. Functionally, upregulation of miR-181a-5p remarkably inhibited the proliferation, migration, and invasion of RB cells and induced apoptosis. Conversely, inhibition of miR-181a-5p yielded the opposite result, suggesting that miR-181a-5p plays a role in suppressing RB progression. miR-181a-5p plays different roles in different cancers. For example, in comparison with that in normal cervical epithelial cells, *i.e.*, End1/E6E7, the expression of miR-181a-5p was remarkably upregulated in cervical cancer cell lines (HeLa and SiHa), while inhibition of miR-181a-5p upregulates the expression of Inositol Polyphosphate-5-Phosphatase A (INPP5A), thereby markedly reducing the proliferation and invasion of cervical cancer cells.^14^ A significant increase in miR-181a-5p was observed in multiple leukemia cell lines and acute lymphocytic leukemia samples, and miR-181a-5p activates the Wnt/β-catenin signaling pathway by inhibiting WIF1, thus promoting leukemia cell growth.^15^ These oncogenic properties of miR-181a-5p in these diseases are contrary to the findings of this study. However, in hepatocellular carcinoma tissues, miR-181a-5p is markedly downregulated; by targeting c-Met, miR-181a-5p represses the proliferation and invasion of cancer cells.^16^ Similarly, miR-181a-5p expression is remarkably downregulated in bladder cancer tissues and cell lines, and *in vitro* experiments have shown that miR-181a-5p overexpression suppresses the proliferation, migration, and invasion of bladder cancer cells.^17^ These reports focusing on miR-181a-5p are consistent with the results of the present study. These studies suggest that the biological function of miR-181a-5p is distinct in different cancers, depending on its target genes.

One of the most common ways for miRNAs to exert their biological functions is to affect post-transcriptional translation by specifically binding to the 3′-UTR of downstream target mRNA. In the present study, the authors predicted and confirmed that NRAS is a direct target of miR-181a-5p. Further research revealed that overexpression or knockdown of NRAS reversed the effects of overexpression or inhibition of miR-181a-5p on RB cells. These findings indicate that miR-181a-5p exerts its role in inhibiting RB development, at least to some extent, by downregulating NRAS. Several studies have reported the cancer-promoting function of NRAS in different cancers, including RB. For example, NRAS knockdown sensitizes cisplatin-resistant GC cells to cisplatin.^18^ In hepatocellular carcinoma, NRAS expression is markedly upregulated in hepatocellular carcinoma tissues and cell lines; the combined inhibition of KRAS and NRAS reduces the resistance of hepatocellular carcinoma to sorafenib.^19^ These studies suggest that targeting NRAS is expected to become a promising strategy for cancer treatment. It has been reported that NRAS knockdown represses the proliferation, migration, and invasion of RB cells,^10^ which is consistent with the present study's results. The detailed mechanism by which NRAS mediates RB progression has not yet been clarified. Interestingly, some studies have reported that some downstream signaling pathways of NRAS, such as MAPK^20^ and PI3K/Akt^21^ pathways, are involved in the regulation of the malignant biological behaviors of RB cells. Whether NRAS promotes RB progression by regulating these pathways and the regulatory functions of miR-181a-5p on these pathways, remain to be explored in future studies.

In summary, through *in vitro* assays, the authors validated that miR-181a-5p expression is significantly downregulated in RB, and it can target NRAS to impede the proliferation, migration, and invasion of RB cells while promoting apoptosis. The present research further elucidates the molecular mechanism underlying the development of RB and suggests that miR-181a-5p may be a promising therapeutic target for RB.

## Authors' contributions

Ouyang M conceived and designed the experiments. Ouyang M, Xiong C, and Rao J performed the experiments. Liu G and Xiong C performed the statistical analysis. Ouyang M and Liu G wrote the manuscript. All of the authors read and approved the final version of the manuscript.

## Conflicts of interest

The authors declare no conflicts of interest.
